# Impact of the COVID-19 pandemic on the quality of life and accessing rehabilitation services among patients with spinal cord injury and their fear of COVID-19

**DOI:** 10.1186/s13018-023-03804-7

**Published:** 2023-04-25

**Authors:** Fater A. Khadour, Younes A. Khadour, Bashar M. Ebrahem, Ling Meng, Cui XinLi, Tao Xu

**Affiliations:** 1 Department of Rehabilitation, Tongji Hospital, Tongji Medical College, Huazhong University of Science and Technology, 1095#, Jie-Fang Avenue, Qiaokou District, Wuhan, 430030 Hubei China; 2 Department of Rehabilitation, Faculty of Medicine, Al Baath University, Homs, Syria; 3 Physical Therapy Department for Neuromuscular and Neurosurgical Disorder and Its Surgery, Cairo University, Cairo, 11835 Egypt; 4 Department of Sport Education, Neijiang Normal University, Sichuan, 641004 China

**Keywords:** Spinal cord injury, COVID-19, Quality of life, Pandemics, Fear

## Abstract

**Background:**

The unanticipated coronavirus disease (COVID-19) had a negative effect on the quality of life (QoL) of patients with spinal cord injury (SCI) and made significant changes in their daily routine. Patients with SCI face additional health risks, especially mental, behavioral, and physical. Without regular physiotherapy sessions, patients' psychological and functional abilities can deteriorate, and complications can occur. There is little information available about the impact of COVID-19 on the quality of life of patients with SCI, and their access to rehabilitation services during the pandemic.

**Objective:**

This study was designed to examine the effects of the COVID-19 pandemic on the quality of life of patients with SCI and also their fear of COVID-19. The pandemic’s impact on the accessibility of rehabilitation services and attendance at physiotherapy sessions in one Chinese hospital were also documented.

**Design:**

An observational study based on an online survey.

**Setting:**

Outpatients clinic at the rehabilitation department of Wuhan's Tongji Hospital.

**Participants:**

People who had been diagnosed with a spinal cord injury (SCI) and who were receiving regular medical monitoring as outpatients at the rehabilitation department were invited to participate in our study (*n* = 127).

**Intervention:**

Not applicable.

**Outcome measures:**

A 12-Item Short-Form Health Survey (SF-12) designed to measure participants' quality of life before and during the pandemic. Their fear of COVID-19 was quantified using the Fear of COVID-19 Scale (FCV-19S). Demographic and medical status information was extracted from their medical records. Their use of rehabilitation services and attendance at physical therapy sessions was also documented.

**Results:**

Seventy-nine patients with SCI completed the SF-12 and FCV-19 scale. The mental and physical aspects of the participants’ quality of life declined significantly, during the epidemic compared to the pre-epidemic period. More than half of the participants have experienced fear of COVID-19 based on FCV-19S. Most received only irregular physical therapy during routine checkups. Worry about virus transmission was the most common cause cited for not attending regular physical therapy sessions.

**Conclusions:**

The quality of life of these Chinese patients with SCI declined during the pandemic. Most of the participants were shown a high level of fear of COVID-19 and were classified as having an intense fear of COVID-19, in addition to the impact of the pandemic on their access to rehabilitation services and attendance at physical therapy sessions.

## Introduction

In late 2019, the coronavirus COVID-19 emerged in Wuhan, China [1]. In March 2020, COVID-19 infection was upgraded to a pandemic because more than 10,000 cases of COVID-19 infection had been detected around the globe. The rapid increase in infection had the potential to deplete essential hospital supplies and overburden health and medical systems [2].

The pandemic affected different aspects of life, such as health and education, and resulted in to suspend of most of the academic institutions where in-person educational activities, including face-to-face teaching and simulation laboratories, have been interrupted along with an interruption of the clinical rotation within the different areas of the same institution [3].

The COVID-19 disease itself and health measures such as quarantine, lockdown, and their subsequent effects such as losing a job, financial difficulties, and impacted daily activities would be expected to have a negative effect on life satisfaction, well-being, and mental health [4].

The impacts of the COVID-19 pandemic have affected all groups in society due to limited freedom, long periods of loneliness, and family separation, which have detrimental repercussions on the psychological status, mental health, and quality of life (QOL) of the general population [5]. QOL can be defined as "the degree of need and satisfaction within the physical, psychological, social, activity, material, and structural area" of one's life [6]. QOL reflects an individual's level of satisfaction with his or her physical, psychological, and social aspects, as well as the range of challenges they may experience within these domains.

In a survey conducted among Chinese population during the initial period of COVID-19 showed moderate-to-high levels of anxiety, apprehension, and fear associated with low levels of QOL [7]. Another study conducted in the Kingdom of Saudi Arabia reported that the COVID-19 pandemic and repeated lockdown measures had significant effects on QOL, especially for those who experienced depression, anxiety, and chronic diseases [8].

Several studies have shown that people with chronic diseases such as spinal cord injury (SCI) are at risk for lower QOL [9]. SCI can cause various complications and comorbidities, making patients experience lower QOL than healthy controls [10–12].

Moreover, the repeated closures of the cafes, restaurants, and cinemas, since they are considered contamination sites, have led to the restriction of outdoor activities for those patients as well as many countries with a large number of COVID-19 patients, such as Portugal, New York, and Saudi Arabia, have suspended several healthcare disciplines, like physiotherapy centers and rehabilitation services to reduce the spread of infection among patients [13–15]. The need regular physiotherapy sessions and special treatment which became more difficult to access. In Wuhan, for example, many outpatient departments and rehabilitation centers slowed down their activities in response to the social distancing measures designed to minimize transmission of the virus [16]. For individuals with SCI, lack of access to rehabilitation services and physiotherapy outpatient clinics may negatively impact their psychological well-being, QoL, or even their mental health [17]. In contrast, dedicating more time to physical activities and performing therapeutic exercises may enhance well-being and QOL among patients with SCI [18, 19].

Concerns about infection and the rising number of people infected lead to increasing fear of the virus, which in turn leads to increased anxiety, depression, and stress [2, 20]. That there is no credible prediction of how long the epidemic will last contribute to feelings of uncertainty [21]. Recently, several studies have provided evidence confirming these concerns. For example, a survey of 52,730 individuals in China revealed that about one-third had some degree of psychological distress [22].

The high incidence of mortality due to COVID-19 has given rise to a new medical condition: exaggerated fear of COVID-19 [20].

Fear is considered among the pandemic’s most prominent mental health impacts [23]. This may be fear of infection, fear of coming into contact with infected people, fear of transmitting the infection to others, the fear engendered by the repeated emergence of new strains of the virus, or some combination [20, 24].

The pandemic's impacts and psychological stress on people with an SCI have been studied [9, 25, 26], but fears of a pandemic have yet to be evaluated with that group explicitly. In addition, there is not enough research addressing the impact of COVID-19 on the QOL of patients with SCI and their access to rehabilitation services during the pandemic, especially in China.

This observational study was therefore designed to examine the effect of the pandemic on the quality of life (QoL) of patients with SCI and their fear of COVID-19. In addition to investigating the impact of the COVID-19 pandemic on access to rehabilitation services and attendance at physiotherapy sessions.

## Methods

### Recruitment of participants

Participants were recruited according to the inclusion criteria for the study. This was determined as a confirmed clinical diagnosis of SCI and required admission at the rehabilitation department of Wuhan's Tongji Hospital for rehabilitation intervention before the epidemic. All SCI patients admitted for rehabilitation in Tongji Hospital were invited to participate in this survey study. The exclusion criteria included patients with SCI who did not accept participating in this study and who were not admitted for rehabilitation before the epidemic and anyone aged less than 18 years.

According to the mentioned period, a total of 127 patients were invited to participate, and the response rate was 101 (79.5%). Twenty-two patients were excluded according to exclusion criteria (*N* = 10 patients aged less than 18 years, and *N* = 12 patients did not admit for rehabilitation before the pandemic). Finally, only 79 (78.2%) were recruited for this study. From April 15 and May 15, 2021, two nurses collected and documented the data.

### Data collection

Before filling out the questionnaire, all the participants got a brief introduction explaining the aim of the research, the procedures, and how the dataset would be used. They were required to answer all of the questions before they could submit their survey, which ensured no missing data. Participation was voluntary and self-selected, and the respondents received no compensation for participating.

Demographic information of the participants and data about their access to rehabilitation services during the epidemic and any history of COVID-19 infection were collected from their medical records.

In addition, data were collected about the respondent's quality of life and fear of the pandemic. The data were gathered between April 15 and May 15, 2021, but the respondents were asked to complete the questionnaire with reference to the period from January 15, 2020 ,to April 15, 2021. From February 1 to April 1, 2020, outpatient rehabilitation services at the hospital had been suspended.

Of particular interest were the number of a respondent's appointments at the rehabilitation department, as well as the causes for those appointments and any irregular follow-up visits. If there were unscheduled visits, the causes were recorded from the chart review of the participants. Whether the subject had gone to the hospital for any cause between January 15, 2020, and April 15, 2021 was carefully recorded. This study was approved by the ethics committee of Tongji Hospital, Tongji Medical College, Huazhong University of Science and Technology.

### Quality of life

The respondents' QoL Before and during the pandemic was quantified using a Chinese version of the 12-Item Short-Form Health Survey (SF-12) [27]. It consists of 12 items and has a mental component summary (MCS) and a physical component summary (PCS) [27].The participants' QoL before the pandemic was with reference to the period between August 20, 2019, and January 15, 2020, while the SF-12 results during the lockdown were with reference to the period between January 15, 2020, and April 15, 2021.

### Fear of COVID‑19

A Chinese Fear of COVID-19 Scale (FCV-19S) was used to evaluate the respondents' fear of COVID-19 [28]. This is a short, unidimensional instrument that is simple to implement, easy to understand, and appropriate for all ages and genders. The response to each of the scale's five questions was given on a scale ranging from 1 to 5, giving an overall score ranging between 7 and 35 with higher values indicating more fear. This scale has been translated into several languages besides Chinese [28–32]. The Chinese version has demonstrated good psychometric properties [28].

A COVID-19 fear cutoff point of 16.5 was defined such that scores above that level were categorized as indicating "intense fear" [33].

### Statistical analyses

Data analyses were carried out using version 25.0 of the SPSS software suite. The variables were investigated using a Kolmogorov–Smirnov test. In reporting descriptive statistics, normally distributed continuous data were expressed as mean ± standard deviation (SD), while the non-normally distributed continuous data were presented as the median (25th, 75th percentile). Categorical and nominal variables were presented as frequencies and percentages (%). The responses related to QoL before and during the epidemic were compared using paired-sample t-tests. The Pearson coefficient analysis was computed to analyze the significance of any associations between change in QoL and FVC-19S score. Univariable analysis was computed to analyze the significance of any associations between the characteristics of the patients, access to rehabilitation services during the pandemic and FVC-19S score. Variables with significant univariable effects were further processed using logistic multivariate regression analysis. Odds ratio (OR) is presented with a 95% confidence interval (CI). Microsoft Excel (Microsoft Office Package 13) was used for building graphs and tables. The level of significance was fixed at 0.05.

## Results

The demographic features of SCI people are shown in Table 1. The respondents ranged in age from 18 to 84 with a mean age of 40.06 ± 14.52 years. Furthermore, the 30–39-year age group had the largest 26 (32.9%), followed by 40–49 years 22 (27.8%). Most 46 (58.2%) were thoracic, with another 31 (39.2%) cervical. According to ASIA grade (American Spinal Injury Association Scale (AIS) Grade), forty-one of the respondents (51.9%) had suffered complete injuries (ASIA grade A, at cervical, thoracic or lumbar level) with the injuries of the other 38 (48.1%) incomplete (ASIA B, C, D). Traffic accidents were the most common cause 30 (38.0%), followed by falls 25 (31.6%) and work accidents 13 (16.5%).

**Table 1:** Participant demographics

	N	%
Age (years)		
18–29 year	17	21.5
30–39 year	26	32.9
40–49 year	22	27.8
50–59 year	10	12.7
60 years and above	4	5.1
Gender		
Male	68	86.1
Female	11	13.9
Level of injury		
Cervical	31	39.2
Thoracic	46	58.2
Lumbar	2	2.5
ASIA		
A	41	51.9
B	17	21.5
C	19	24.1
D	2	2.5
Completeness of the injury		
Complete	41	51.9
Incomplete	38	48.1
Cause of SCI injury		
Fall	25	31.6
Traffic accident	30	38.0
Work accident	13	16.5
Sport Injury	4	5.1
Non-traumatic injury	7	8.9
Time since injury		
1–5 year	61	77.2
6–10 year	14	17.7
11–15 year	4	5.1
16–20 year		

*N* = 79, *ASIA*: American Spinal Injury Association Scale (AIS) Grade

Seventy-five of the participants (94.9%) made at least one irregular visit. Worry about virus transmission was the most common cause reported for not attending physical therapy sessions. Only 10 of those participants (12.7%) did not interrupt their physiotherapy sessions, whereas 50 (63.3%) took a break from regular physiotherapy sessions. Nineteen (24.1%) stopped going to their physiotherapy sessions completely during the pandemic. Thirteen of the participants (16.5%) reported having gone to the hospital for various other reasons, of which urinary and respiratory system were the most common. More details are shown in Table 2.

**Table 2:** Participants' rehabilitative activities during the pandemic

	*N* (%)
**Outpatient rehabilitation clinic visits (Irregular follow-up)**	
Yes	75 (94.9%)
No	4 (5.1%)
Reason for irregular follow-up	
Fear of catching the virus	51 (64.6%)
Problems of scheduling	13 (16.5%)
Health problems	4 (5.1%)
Transportation problems	5 (6.3%)
Other	2 (2.5%)
Physical therapy attendance during the pandemic	
Continuous	10 (12.7%)
Took a break	50 (63.3%)
Discontinued	19 (24.1%)
Access to other clinics during the epidemic	
Yes	13 (16.5%)
No	66 (83.5%)
Reason for visiting the hospital	
Bowel problems	–
Urinary problems	8 (10.1%)
Respiratory system problems	4 (5.1%)
Skin problems	1 (1.3%)
Others	–
History of COVID-19	
Yes	3 (3.8%)
No	76 (96.2%)
FCV-19S	20.27± (2.6)*

*Mean ± standard deviation; *N* = 79

The mean FCV-19S score was 21 ± 2.6, and 69 of the respondents (87.3%) were classified as having an intense fear of COVID-19. Three of the respondents (3.8%) reported having been infected with COVID-19 (Table 2).

**Fig. 1:**
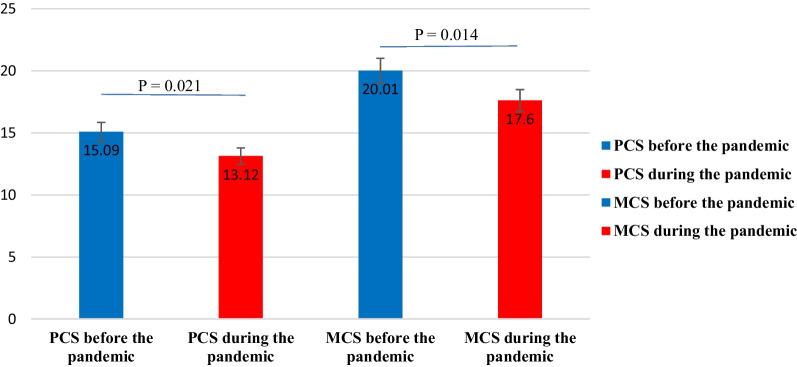
Changes in the participants' quality of life (MCS) is the mental component summary, and (PCS) is the physical component summary

During the epidemic, the mental component summary (MCS) = 17.50 ± 1.29 and the physical component summary (PCS) = 13.12 ± 1.13. This compares to the pre-epidemic period with the mental component summary (MCS) = 20.01 ± 1.38 and the physical component summary (PCS) = 15.09 ± 1.17. Participants' mental and physical QoL dropped significantly during the pandemic compared to the pre-pandemic period (*P* = 0.014, *P* = 0.021 respectively) Fig. 1.

Table 3 shows that there was no significant association between the FCV-19S results and gender of participants, cause of SCI injury, the level of injury or time since injury. They were also not significantly related to the changes in PCS-12 or MCS-12 scores (Table 4). There were, however, significant relationships between the FCV-19S results and the completeness of their injury (*p* = 0.017) and their ASIA score (*p* = 0.019).

**Table 3:** Correlations between the characteristics of participants and FCV-19S

Fear of COVID-19 Scale			
	*N* (%)	*P*-value	OR (95% CI)
Age (years)			
18–29 year	17 (21.5%)	0.06	
30–39 year	26 (32.9%)		
40–49 year	22 (27.8%)		
50–59 year	10 (12.7%)		
60 years and above	4 (5.1%)		
Gender			
Male	68 (86.1%)	0.91	
Female	11(13.9%)		
Level of injury			
Cervical	31(39.2%)	0.78	
Thoracic	46 (58.2%)		
Lumbar	2 (2.5%)		
ASIA			
A	41(51.9%)	0.019	
B	17 (21.5%)		
C	19 (24.1%)		
D	2 (2.5%)		
Completeness of the injury			
Complete	41 (51.9%)	0.017	1.75 (0.96–3.11)
Incomplete	38 (48.1%)		1.00
Cause of SCI injury			
Fall	25 (31.6%)	0.46	
Traffic accident	30 (38.0%)		
Work accident	13 (16.5%)		
Sport Injury	4 (5.1%)		
Non-traumatic injury	7 (8.9%)		
Time since injury			
1–5 year	61 (77.2%)	0.83	
6–10 year	14 (17.7%)		
11–15 year	4 (5.1%)		
> 15 years	–		

*N* = 79, *ASIA*: American Spinal Injury Association Scale (AIS) Grade. The odds ratio (OR) is presented with a 95% confidence interval (CI)

**Table 4:** Correlations between FCV-19S scores and patients’ QOL

	Change in MCS -12 score	Change in PCS -12 score
Results of FCV-19S		
P	0.99	0.139
r	0.38	0.22

PCS-12: Physical Component Summary of Short-Form-12; MCS-12: Mental Component Summary of Short-Form-12; FCV-19S: Fear of COVID-19 Scale, r: Spearman's rho correlation coefficient

Additionally, there was no significant association between the FCV-19S results and access to the hospital or clinics during the pandemic, but the association with attendance at physiotherapy sessions (*p* = 0.03) and irregular follow-up visits was significant (*p* = 0.004) (Table 5).

**Table 5:** Correlations between rehabilitative activity and FCV-19S

Fear of COVID‑19 scale			
	*N* (%)	*P*-value	OR (95% CI)
Outpatient rehabilitation clinic visits (irregular follow-up)			
Yes	75 (94.9%)	0.004	1.64 (1.12–2.31)
No	4 (5.1%)		1.00
Reason for irregular follow-up			
Fear of catching the virus	51 (64.6%)	0.07	
Problems of scheduling	13 (16.5%)		
Health problems	4 (5.1%)		
Transportation problems	5 (6.3%)		
Other	2 (2.5%)		
Physical therapy attendance during the pandemic			
Continuous	10 (12.7%)	0.03	
Took a break	50 (63.3%)		
Discontinued	19 (24.1%)		
Access to other clinics during the epidemic			
Yes	13 (16.5%)	0.09	
No	66 (83.5%)		
Reason for visiting the hospital			
Bowel problems	–	0.08	
Urinary problems	8 (10.1%)		
Respiratory system problems	4 (5.1%)		
Skin problems	1 (1.3%)		
Others	–		
History of COVID-19			
Yes	3 (3.8%)	0.90	
No	76 (96.2%)		

The odds ratio (OR) is presented with a 95% confidence interval (CI)

Four factors with significant differences in the univariate analysis were further together analyzed by logistic multivariate regression analysis to acquire adjusted OR, including the completeness of their injury, ASIA score, attendance at physiotherapy sessions and irregular follow-up visits. Results revealed that the fear of COVID-19 was more effective on the following two factors, completeness of their injury, and irregular follow-up visits. Patients with complete SCI had more fear of COVID-19 than patients with incomplete SCI (OR 1.75, 95% CI 0.96–3.11). In addition, the fear among the patients during the pandemic affected their attendance at the physiotherapy sessions. The more fear of COVID-19, the more irregular follow-up visits to the outpatient rehabilitation clinic among patients with SCI (OR 1.64, 95% CI 1.12–2.31), (Table 4).

## Discussion

The COVID-19 pandemic has created a considerable challenge to the health system because of the enormous influx of COVID-19 patients into healthcare facilities, often requiring long-term or intensive care. As a result, healthcare facilities, particularly neurological departments, rehabilitation services, and physiotherapy centers that treat SCI patients, have had to make significant changes, such as foregoing many non-urgent elective surgeries and minimizing patient volume.[34]. In addition, many physiotherapy centers have sought to curtail their activities in response to the social distancing procedures intended to reduce virus transmission. [35]. Furthermore, the state of anxiety and fear among the patients prompted many of them to discontinue attending their physiotherapy sessions [36].

This study has revealed that during the COVID-19 pandemic, most of the participants made irregular visits to rehabilitation clinics during routine checkups. Fear of catching the virus was the most commonly reported cause for not attending their physiotherapy sessions. Half of them took a break from regular physiotherapy sessions and stopped going to their physical therapy sessions altogether.

There was, however, no significant relationship between fear of the virus and changes in the participants' physical or mental QOL. Fear of COVID-19 was, however, significantly related to the completeness of a respondent's injury. More complete injury predicts greater fear of COVID-19. And such fear significantly impacted the respondents' hospital visits or their ability to maintain attendance at their physiotherapy sessions. This could be attributed to the patients with SCI who are considered more at risk of infection because of their weakened immune system [21, 23], and their secondary health issues make them more susceptible to getting the virus [37]. These results are in line with a Japanese study conducted among patients with SCI showing that most participants had reduced frequency of going out and receiving home-visit nursing or rehabilitation services during the pandemic [38].

Patients with SCI during the COVID-19 pandemic experienced various physical, psychological, mental, and social challenges, each of which could negatively affect daily functioning and QOL. The results of this study indicated decreased participants' physical or mental QOL during the COVID-19 pandemic than pre-pandemic. The findings align with a previous study showing that persons with chronic diseases are more likely to have lower QOL scores during COVID-19 than pre-pandemics [39]. But the current results were in contrast to a studyconducted in Spain by Rudolf et al. [40], which reported no significant differences in patients with SCI before and during the pandemic across any of the physical and mental dimensions of the World Health Organization's quality of life-BREF (WHOQOL-BREF) domains. However, when participants were stratified into two groups based on their age, the younger group reported significantly lower scores on the physical and psychological dimensions of the WHOQOL-BREF questionnaire scores during the pandemic than they had before. There was no such significant difference among the older group [40].

The daily routines and physical activities of patient with SCI changed due to the pandemic. Another Spanish study of full-time manual wheelchair users with a thoracic SCI between T2 and T12 showed reduced physical activity compared to the pre-epidemic period [41]. With or without a disability, people had to leave school or work, spent more time watching television, and had irregular sleep times and mealtimes [41]. Patient with SCI became more reliant on a caregiver and they too worried about catching the virus. These lifestyle modifications have been shown to induce psychosocial distress in people at greater risk of virus transmission, including patients with SCI [16, 25]. A British study of patients with an SCI which asked open-ended questions about their experiences during the epidemic revealed that most of them had health anxieties which were exacerbated by what the respondents perceived to be their increased vulnerability to respiratory problems and social isolation [42].

In China, discomfort, pain, depression and anxiety were considered the most common problems during the pandemic [22]. Health anxiety in general and fear of COVID-19 in particular have been related to post-traumatic stress symptomatology [20, 24]. Symptoms of anxiety were found in 20.5% of the respondents in China during the pandemic [43] and symptoms of depression were found in 36.4%. That has a detrimental impact on the QoL of patients with SCI [44], as this study has confirmed, where patients with disabilities such as patients with spinal cord injury (SCI), are considered more at risk from COVID-19 infection because of their social and clinical characteristics, like weakend immune system [21, 23]. The most common complication in severe COVID-19 cases is respiratory impairment. Patients with SCI are more vulnerable to respiratory problems [45], which are identical to COVID-19 symptoms such as dyspnea (33%), cough (52%) and fever (74%) [46]. Moreover, they typically suffer from secondary health issues that make them more susceptible to SARS-CoV-2 and can be lethal [37]. This leads to more fear among this group of people.

In addition, COVID-19 has changed the routines of physiotherapy sessions and access to rehabilitation services among patients with an SCI [36]. It has interrupted their rehabilitation sessions, suspending physical therapy and other rehabilitation interventions, which will normally cause a deterioration in mental and physical functioning. Hearn et al. [17] who reported a substantial deterioration regarding the mental and physical aspects of life among patients with SCI. This deterioration be attributed to the lack of access to healthcare facilities and the obligatory isolation due to quarantine.

An important lesson is that in a pandemic vulnerable persons require further education about the importance of appropriate physical activity.

There are some concerns about the health, safety, and protection of patients with SCI in the community [47]. As a result, in this study, an additional aim was to gain a deeper understanding of the pandemic's overall psychological effects on the quality of life of patients with SCI and the effect of the pandemic on their attending of physiotherapy sessions, so that this knowledge might be used to support and guide future care. As well as future studies should focus on how patients with SCI in the community adapt to and cope with continuous waves of the COVID-19 virus and how these influence their livelihood and daily life routine.

## Study limitations

This study's sample size was smaller than it might have been because some potential participants could not be contacted by phone. Others' responses were omitted from the analyses owing to people not matching the inclusion criteria and the small sample size might have affected the results because of a lack of statistical power. In addition, the absence of equality between the proportion of male and female participants causes limited generalizability by the proportion of male participants. The questionnaire-based portion of the study depended on the respondents' memories of their physical and mental QoL during the period studied. Recall bias may therefore have been present in addition to the ever-present tendency for socially acceptable responding.

## Conclusions

The physical and mental QoL of the patients with SCI was significantly degraded during the pandemic. Most of them had a fear of COVID-19 categorized as intense. The pandemic disrupted the rehabilitation programs of many patients with SCI. Patients with complete SCI injuries were most impacted by the COVID-19 pandemic, which affected the continuance of attendance of their physiotherapy sessions. So, more attention should be taken to those patients to avoid staying away from their rehabilitation services since lack of access to healthcare facilities and rehabilitation services substantially deteriorates the mental and physical aspects of life among patients with SCI [17].

## Data Availability

The data generated in this study are available from the corresponding author on reasonable request.
